# Impact of Proteins on the Cellular Uptake of Carbon Nanodots

**DOI:** 10.1021/acsomega.5c09510

**Published:** 2025-12-03

**Authors:** Ziyao Liu, Huijie Yan, I. Jénnifer Gómez, Carolina Carrillo Carion, Blanca Arnaiz, Dingcheng Zhu, Maurizio Prato, Wolfgang J. Parak, Neus Feliu, Michele Cacioppo

**Affiliations:** † Fachbereich Physik, 153609 Universität Hamburg, ChyN, Luruper Chaussee 149, Hamburg 22607, Germany; ‡ Center for Cooperative Research in Biomaterials (CIC biomaGUNE), Basque Research and Technology Alliance (BRTA), San Sebastián PV 20014, Spain; § Centro Interdisciplinar de Química e Bioloxía (CICA), 16737 Universidade da Coruña, Rúa as Carballeiras, A Coruna 15001, Spain; ∥ Institute for Chemical Research (IIQ), CSIC-University of Sevilla, Sevilla 41092, Spain; ⊥ Dipartimento di Scienze Chimiche e Farmaceutiche (DSCF), Università degli Studi di Trieste, Via L. Giorgieri 1, Trieste 34127, Italy; # Basque Foundation for Science, Ikerbasque, Bilbao PV 48013, Spain; ∇ Dipartimento di Scienze e Tecnologie Biologiche Chimiche e Farmaceutiche (STEBICEF), Università degli Studi di Palermo, Viale delle Scienze − Ed. 17, Palermo 90128, Italy

## Abstract

There are many reports in the literature on how a formed protein corona around colloidal nanoparticles affects their uptake by cells. There are however still details to be investigated, such as the role of a preformed protein corona versus the protein corona formed in serum-supplemented culture media, which is the topic of this work. Carbon nanodots (CDs) were preincubated with human serum albumin (HSA), and their cellular uptake was studied with flow cytometry and confocal laser scanning microscopy, in comparison to bare CDs. Measurements were carried out under serum-free and serum-containing conditions, leading to a 2 × 2 sample set: CDs with proteins (HSA preincubation + serum, serum, and HSA preincubation), and CDs without proteins. Serum-supplemented conditions lead to reduced uptake of CDs, whereas preincubation with HSA enhanced their internalization.

## Graphical abstract

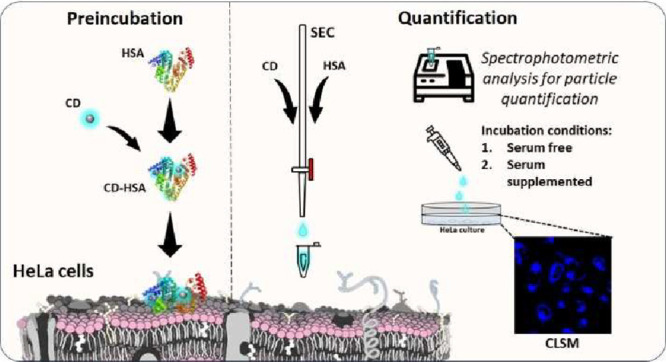

## Introduction

Fluorescent carbon dots (CDs), a prominent class of the carbon-based nanomaterials family, have attracted considerable attention for their potential biomedical applications,^1,2^ including biosensing, cellular imaging, drug delivery, and theranostics.^1,3^ Indeed, CDs exhibit unique optical tunability, high aqueous dispersibility, and low cytotoxicity. Moreover, their ultrasmall size potentially enables biodistributions different from the ones of bigger nanoparticles, which could be advantageous for biomedical applications.^4^ However, their small dimensions also pose analytical challenges, as parameters such as molar mass, surface area, and protein adsorption stoichiometry are difficult to determine precisely. These limitations complicate quantitative correlations between their physicochemical properties and biological responses. In addition, when CDs are introduced into biological fluids, such as cell culture medium or blood, the proteins present in these fluids will instantaneously interact with the CDs. In this context, it is important to distinguish between preformed and in situ evolving coronas. While the latter arises dynamically in complex biological media, a preformed corona offers a controlled model to isolate the influence of protein adsorption kinetics and corona composition on nanoparticle–cell interactions.^5,6^ Such interactions are particularly relevant for small nanoparticles, whose dimensions are comparable to those of individual proteins. Due to their ultrasmall size, a single protein could interact with multiple dots through different binding sites.^7^

In bionanotechnology, this interaction is often related to the so-called process of “protein corona” formation.^5,6^ The basic concept of this process is described by the formation of a shell of proteins on a nanoparticle surface. The expression “protein corona”, however, could be misinterpreted when nanoparticle sizes are so small that on average less than one protein binds per nanoparticle.^4,8,9^ In these cases, one could also argue that proteins are labeled with the nanoparticles, e.g., there is a corona of nanoparticles around the proteins. The interaction of nanoparticles and proteins is important when designing nanosystems for biological applications.^10^ Indeed, this interaction impacts nanoparticles, for example, in different aqueous biological microenvironments.^11^ Another relevant case in this context is the extracellular interaction of nanoparticles with the cellular membrane.^12^ In this case, adsorption of proteins onto the original functionalized surface of the nanoparticles may influence their binding to receptors in the cell membrane.^13,14^

A basic quantifier used to study the influence of protein interaction with nanoparticles is the amount of proteins adsorbed per nanoparticle, which can, for example, be determined with mass spectrometry.^15^ However, this becomes a challenging task when dealing with ultrasmall nanoparticles such as CDs. In such cases, even determining the molar nanoparticle concentration is complicated. While the mass concentration can be measured by weighing a dried nanoparticle sample, the size distribution of the particles makes their molar mass poorly defined, introducing errors in the conversion from mass to molar concentration. Moreover, weighing also accounts for ligands and adsorbed molecules, which are not necessarily included in the nanoparticle molar mass, further complicating the conversion.^4^ However, the peculiar optical properties of CDs could be exploited as a useful tool to make a calibration model to have, at least, an approximate quantification of the protein/CD ratio.^16,17^ While there are thousands of different serum proteins, for basic studies, it is convenient to focus on model proteins. In this context, the naturally abundant human serum albumin (HSA) is considered an *ad hoc* human protein model for such studies, since it presents many functions including high binding abilities and blood transport of different compounds.^18,19^ For instance, several HSA-based drug and nanoparticle formulations have been developed for biological applications.^20,21^ Since it has been found that HSA is mainly affine to interact with hydrophobic or positively charged nanoparticles,^22,23^ we selected nitrogen-doped CDs characterized by a positively charged surface.^24,25^

In this work, the exploitation of cross-spectrophotometric techniques has been approached to quantify the proportion of nitrogen-doped CDs and serum albumins for studying the effects on cytotoxicity and the typical biological interaction of cell uptake. By comparison, these effects could be evaluated when such nanoparticles could be considered “naked” or “carried by proteins”, giving insights into the role of the protein–CD interaction. Cellular uptake has been approached by an in vitro study through the application of CD–protein assemblies to the human cervical cancer HeLa cell line.

## Results and Discussion

To assess the effect of proteins on CD uptake, experiments should include both bare CDs and CDs in the presence of proteins. However, using only CDs does not guarantee that they remain protein-free, as cell culture media contain abundant proteins, approximately 60% of which are albumins.^26^ In this work, serum-supplemented conditions served as a reference for the complex, multiprotein corona, enabling comparison with the defined single-protein system. This design allows the separation of specific effects related to the model protein from those arising from heterogeneous serum interactions.^27^ In more complex biological fluids, distinct serum proteins may compete for nanoparticle binding, potentially giving rise to coronas of different compositions and biological responses. The present single-protein model thus provides a controlled reference framework to interpret such multicomponent interactions. In fact, serum proteins are well-known to possess wide binding abilities toward both charged and uncharged particles or biomolecules.^28,29^ This complicates experimental design as it is difficult to precisely evaluate how CDs interact with such a large variety of proteins. In other words, is there a difference between CDs that are precoated with proteins and those that encounter proteins only when introduced into a protein-containing medium during cell incubation?

### Metrics for the Characterization of the CD-HSA Complexes

The first attempt was to study the composition of the CDs-HSA complexes originating from the preincubation of CDs with HSA (see the sketch in Scheme 1). The CDs have an excitation range between λ_exc_ = 300 and 500 nm, with a maximum of emission at about 355 nm, and their mass concentration *C*_CD_ thus can be quantified via fluorescence intensity measurements (Figure S1). Despite a good linear correlation being obtained at an excitation wavelength of 380 nm (Figure S1d), we opted to select the excitation wavelength of λ_exc_ = 300 nm as the best compromise for a concentration range from *C*_CD_ = 10 to about 300 μg mL^–1^ (Figure S2), since at this value the CDs exhibit their maximum emission (Figure S3c), while the HSA absorption contribution is practically not significant (Figure S4).

**Scheme 1:**
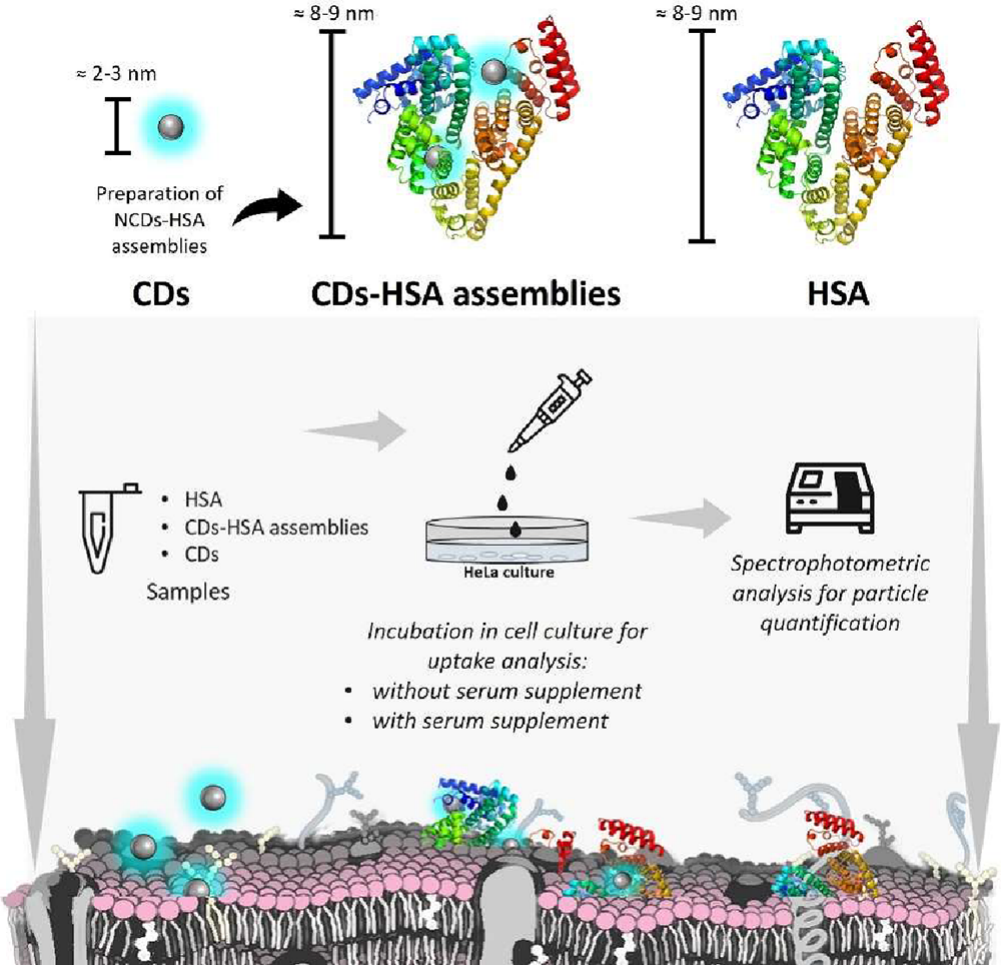
Schematics of the HSA and CD Quantification Methodologies and Cellular Uptake Study

For the quantification of the HSA concentration of *C*_HSA_, two different approaches were used. First, the concentration of unlabeled HSA was determined with the Brandford assay at 595 nm absorbance.^30,31^ Alternatively, fluorescence-labeled HSA was used. For this, an indolenine based dye (Cy-7 or Cy-5 in the form of sulfo-cyanine NHS ester, from now on called “Cy”) was selected as the label for HSA (forming HSA-Cy, see the Supporting Information – Materials and methods, for experimental details and protocol), since it presents fluorescence emission with a peak at 773 nm (see Figure S3d). In this way, HSA-Cy and CD in CDs-HSA-Cy complexes can be quantified separately by fluorescence emission. However, the labeling of HSA presents issues such as the structure and the overall charge modification that could affect the binding activity toward the CDs.^32^

For obtaining precoated CDs-HSA-Cy (or CDs-HSA), HSA-Cy (or HSA) was incubated with an excess of CDs, with a CDs/HSA molar ratio c_CD_/c_HSA_ of about 15 to 7000. Molar ratios were calculated by adjusting the masses of CDs and HSA-Cy to prepare the mixtures and their respective molecular weights (*M*_CDs_ = 1000 g/mol, *M*_HSA_ = 66,000 g/mol^33^). Atomic force microscopy (AFM) analysis revealed that the obtained CDs-HSA-Cy aggregates were larger than individual CDs (Figure S5). In support of the AFM data, additional characterization by DLS (Figure S5d), fluorescence spectroscopy (Figures S3 and S4), and size exclusion chromatography (Figures S6–S8) confirmed the reproducible formation of CD-HSA supramolecular assemblies. The comparable spectrophotometric profiles observed in three independent SEC preparations further demonstrate the reproducibility of these assemblies (Figures S6–S8). For small CDs, whose diameter (<3 nm) is considerably smaller than that of most serum proteins such as HSA (ca. 7 nm), the classical concept of a nanoparticle–protein corona becomes less applicable. Instead, the interaction leads to the formation of protein–nanoparticle adducts, where the nanoparticle binds to the exposed domains on the protein surface rather than being completely covered by a multilayer corona. This model would explain the moderate increase in hydrodynamic diameter observed in Figure S5 and is consistent with previous reports on small nanoparticles showing size-dependent deviations from conventional corona behavior.^34–37^ It is also known that adsorption of nanoparticles onto proteins can induce local rearrangements or partial unfolding of their secondary structure depending on nanoparticle size, surface chemistry, and the relative particle-to-protein ratio. In our experiments, after purification, the CD/HSA assemblies displayed an excess of CDs relative to HSA (see Tables S1 and S2), suggesting that only a limited fraction of the protein interacts with the CD surface. Under such conditions, major conformational changes in HSA are unlikely. This interpretation is supported by previous studies reporting that nanoparticle–protein interactions may lead to conformational variations that remain moderate when the protein content is relatively low or when adsorption occurs through limited surface contacts.^38,39^

In order to separate free HSA from the complexes, these formulations were further purified by size exclusion chromatography (PD-10 column). In the PD-10 column, macromolecules such as HSA elute in the first volume, while smaller particles such as CDs penetrate the gel pores and elute later. This elution pattern enables a qualitative distinction between free proteins and nanoparticle–protein assemblies.^34,35^ We note that upon removal of unbound protein, the protein–nanoparticle equilibrium is changed, which may lead to the desorption of some originally adsorbed proteins from the nanoparticles.^34^ However, it will be assumed that there is a minor error in comparison to other error sources. The different fractions of the eluted reaction mixture of CDs and HSA-Cy (or HSA) were collected, together with CDs and HSA-Cy (or HSA) only as controls (see Figures S6–S8). For unlabeled HSA, the control shows that HSA elutes prior to CDs (Figures S6 and S7). The eluted fractions were pooled to two final samples, and the CD/HSA molar ratio *c*_CD_/*c*_HSA_ was calculated by the Bradford assay (for the conversion between mass and molar concentrations of *C*_CD_ and *c*_CD_, we refer to the SI, Table S1). The first eluate, “fraction A”, contains both free HSA and CDs-HSA with *c*_CD_/*c*_HSA_ ≈ 100 (Table S2). The second elute, “fraction B”, contains free CDs and CDs-HSA with *c*_CD_/*c*_HSA_ ≈ 3. It clearly needs to be stated that with this separation technique, it was not possible to isolate pure fractions of CDs-HSA, and always predominantly, either free HSA or free CDs will be present as a byproduct. In previous work, using fluorescence correlation spectroscopy (FCS), we estimated an equilibrium ratio of *c*_CD_/*c*_HSA_ ≈ 0.5–1, see the Supporting Information of Zhu et al.^21^ While in FCS, the *c*_CD_/*c*_HSA_ ratio of CDs-HSA can be measured disregarding the presence of free HSA; in our present work, the determined *c*_CD_/*c*_HSA_ ratios always contained CDs-HSA as well as unbound CDs or HSA (the Bradford assay does not allow one to distinguish between bound and unbound HSA). Due to the high CDs/HSA ratio *c*_CD_/*c*_HSA_ = 100 set for the reaction, the obtained high CDs/HSA ratio is not surprising. We also refer to the work of others regarding high NP/HSA ratios for ultrasmall NPs.^40–44^

### Uptake of CDs-HSA-Cy versus CDs by Cells

A quantitative analysis of the uptake of CDs in HeLa cells was carried out using fluorescence-based flow cytometry. For the CDs preincubated with HSA, different CD:HSA ratios were used, which were not purified by size exclusions chromatography. Data show a concentration-dependent uptake of both CDs and CDs-HSA, under both serum-free and serum-supplemented conditions (Figure 1). The presence of serum (Figure 1a) reduced CD uptake compared to serum-free culture (Figure 1b), though the effect was less pronounced than that in some previous studies, where much higher NP uptake in serum-free versus serum-supplemented media has been reported.^4,45^ This variation lies within the experimental uncertainty and should be interpreted as a qualitative trend rather than a statistically significant difference. For small CDs, the limited effect of serum is likely related to their small hydrodynamic size and weakly bound corona.^4,9,23,46^ We hypothesize that particle size governs the extent to which corona formation controls cellular uptake.

**Figure 1:**
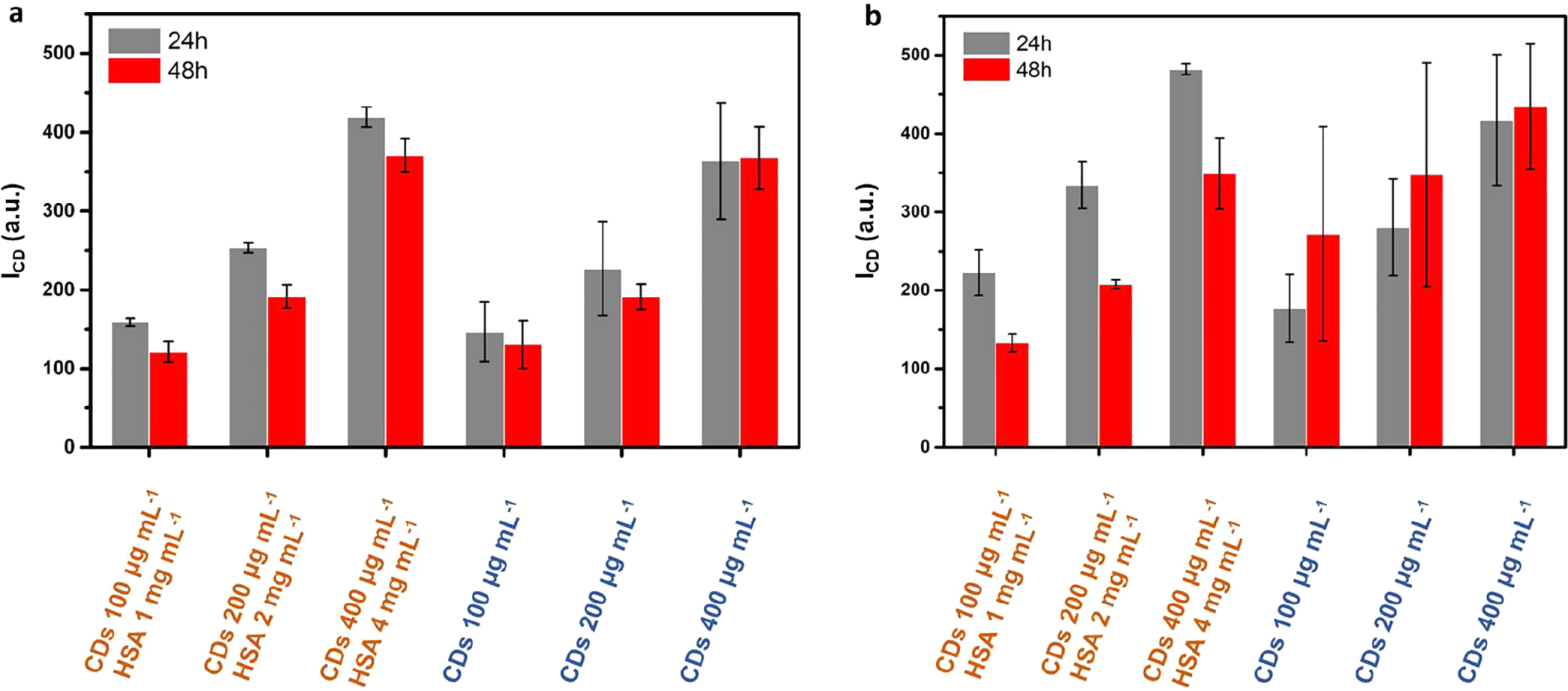
Flow cytometry analysis of cellular uptake using freshly mixed CDs and HSA (CD/HSA preincubation mass ratio 1:100, without size exclusion chromatography purification). This mixture contains both CD-HSA complexes and free HSA. Bare CDs were used as controls. The fluorescence intensities I_CD_ recorded in cells are given for HeLa cells with (a) and without (b) FBS supplement after 24 or 48 h of incubation.

It is likely that the absence of serum had a negative effect on the cells during the 48 h incubation, as the uptake after 48 h was significantly lower than that after 24 h, unlike the trend observed in serum-supplemented medium. Therefore, the 48 h data are not considered for further interpretation. In the case of the 24 h data, there is more uptake for CDs-HSA versus CDs under serum-supplemented and serum-free conditions. It has been reported that there can be a protein corona even on protein NPs;^23,46^ thus, there may be an additional protein corona formed on CDs-HSA under serum-supplemented conditions.

Subsequently, another analysis technique was employed. In contrast to the above-discussed flow cytometry experiments, in which only the amount of fluorescent CDs *per* cell is evaluated per s*e*, microscopy data allow for lateral resolution, e.g., the intracellular location of the materials can be seen. In Figures S9–S14, confocal laser scanning microscopy (CLSM) images of HeLa cells are shown, which have been exposed to CDs, CDs-HSA, or CDs-HSA-Cy. The CLSM images reveal the internalization of both CDs and CDs-HSA under serum-free and serum-containing conditions (Figures S9–S13). The CDs were able to enter cells via endocytosis without the need for additional functionalization, and no significant differences in intracellular localization were observed between bare CDs and CDs-HSA-Cy (Figure S14), both exhibiting the characteristic punctate pattern typical of endocytosed nanoparticles. Although colocalization experiments with specific vesicular markers would be required to confirm this pathway, observations are in agreement with the general finding that nanoparticles of all sizes are endocytosed (which can happen with different pathways).

Cells were incubated with CDs for 24 h, and the resulting intracellular fluorescence intensity I_CD_ was measured by CLSM using two fractions obtained via size exclusion chromatography (i.e., “fraction A” and “fraction B”). Higher uptake under serum-free (“–FBS”) versus serum-supplemented (“+FBS”) culture conditions was observed for CDs and CDs-HSA-Cy (Figure 2). There was higher uptake for CDs-HSA-Cy than for CDs (Figure 2). Both findings are similar to the flow cytometer data (Figure 1). Surprisingly, within the error margins, there was little difference found between “fraction A” and “fraction B”, though there was a tendency of less signal of internalized CDs for “fraction A” versus “fraction B”.

**Figure 2:**
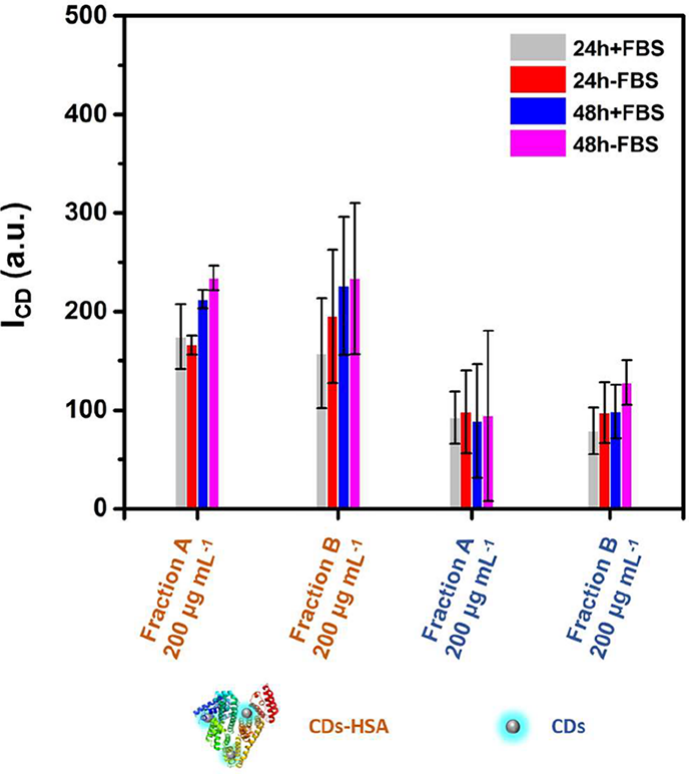
Concentration-dependent uptake study of CDs-HSA assemblies (CD/HSA preincubation mass ratio 1:100) and CDs after purification by size exclusions chromatography (leading to “fraction” A and “fraction” B) by HeLa cells using confocal fluorescence microscopy. Fluorescence intensity of CDs (I_CD_) is reported for serum-supplemented (“+FBS”) and serum-free (“–FBS”) conditions. An additional graph containing these and extra data is shown as Figure S15. For the CD sample, the same elution parameters were used as for the CDs-HSA sample; thus, “fraction A” and “fraction B” were also obtained, despite the fact that there was no HSA in the solution.

An additional experiment was carried out using HSA-Cy instead of HSA for preincubation of the CDs. In this case, apart from the intracellular fluorescence of the CDs (I_CD_), the intracellular fluorescence of HSA-Cy (I_Cy_) was also recorded with CLSM. In agreement with the data shown in Figure 2, higher intracellular intensity I_CD_ was observed under serum-free compared to serum-supplemented conditions, and for CDs-HSA-Cy compared to bare CDs (Figure 3). However, in contrast to the data shown in Figure 2, due to the Cy-labeling of HSA in the data shown in Figure 3, a clear difference between “fraction A” and “fraction B” can be seen. Much higher intensity for HSA-Cy is recorded for “fraction A” than for “fraction B”. According to the size exclusion chromatography results described above, “fraction A” contains free HSA-Cy and CDs-HSA-Cy, whereas “fraction B” contains free CDs and CDs-HSA-Cy. Based on this, the intracellular fluorescence I_CD_ observed after incubation with “fraction B” likely originates primarily from the CDs themselves, suggesting minimal cellular uptake of CDs-HSA-Cy, as there is almost no fluorescence I_Cy_ of “fraction B”. In contrast, the fluorescence seen after incubation with “fraction” A is mainly attributed to CDs-HSA-Cy, as there is fluorescence in both the CD and Cy channels, although in the Cy channel a contribution from unbound HSA-Cy cannot be ruled out.

**Figure 3:**
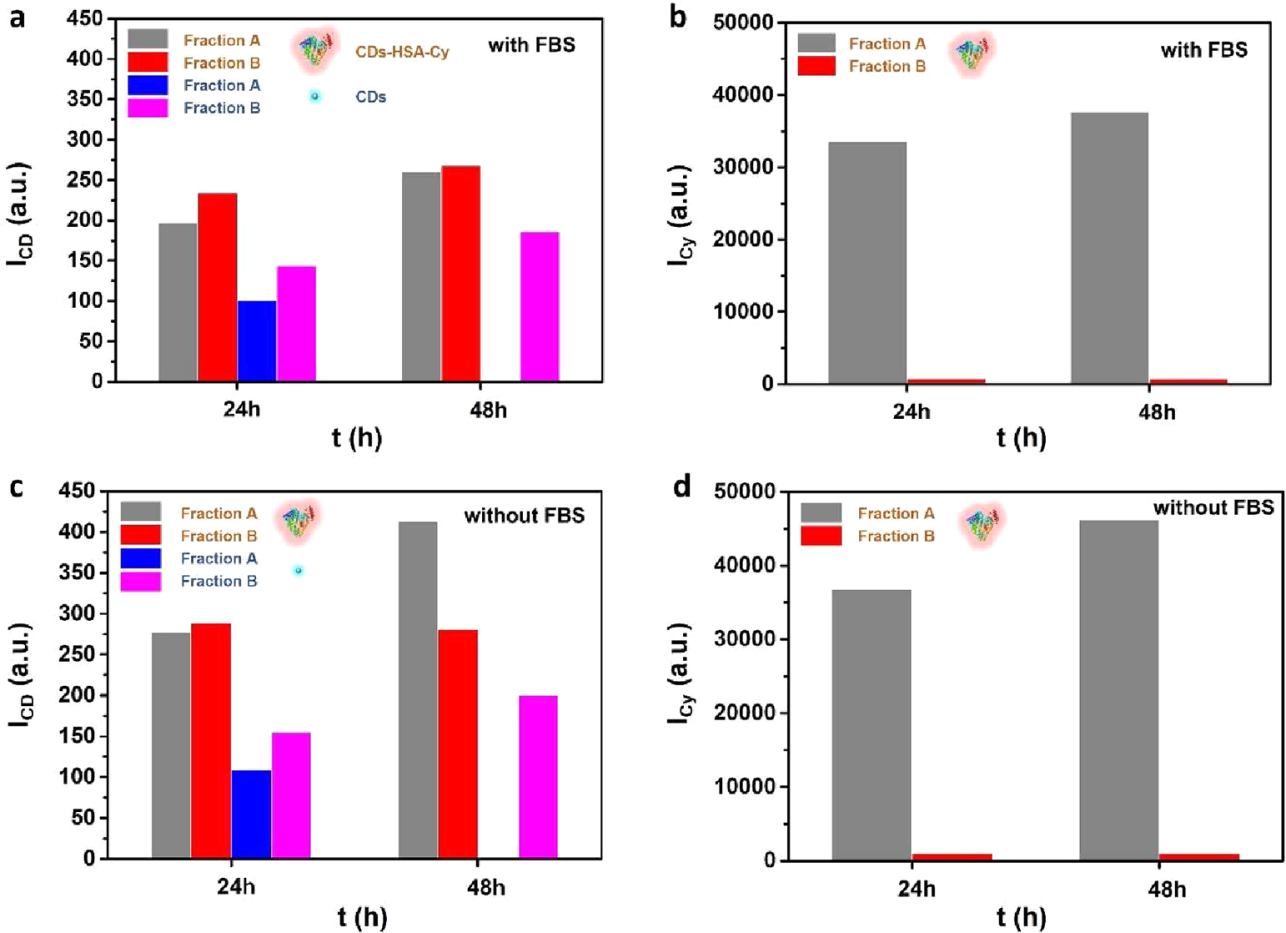
Uptake study of CDs-HSA-Cy and CDs (sorted into “fraction A” and “fraction B”) by HeLa cells using CLSM. Fluorescence intensity of CDs (I_CD_ – a,c) and of HSA-Cy (I_Cy_ – b,d) were detected under serum-free (“without FBS”) and serum-supplemented (“with FBS”) conditions after 24 and 48 h incubation. The 48 h data for fraction A were not recorded due to decreased cell viability after prolonged incubation.

While this study focuses on in vitro mechanisms, in vivo reports suggest that precoating of ultrasmall nanomaterials with albumin can influence their colloidal stability and biodistribution compared with coronas formed directly in serum. Such observations reinforce the relevance of studying preformed protein–nanoparticle assemblies as simplified yet predictive models to understand how controlled surface conditioning may determine subsequent biological interactions. For CDs and other ultrasmall soft nanomaterials, albumin precoating has been linked to modified circulation and organ distribution.^47^

## Conclusions

In summary, this study reveals the distinct biological behavior of preformed versus in situ protein coronas on carbon nanodots (CDs). While serum proteins generally attenuate nanoparticle internalization, preincubation with HSA enhances cellular uptake under both serum-free and serum-supplemented conditions. These findings underscore the kinetic and compositional differences between preadsorbed and dynamically formed coronas^5,6,34^ and highlight the influence of specific protein interfaces on nanoparticle trafficking.

The work further demonstrates that even for ultrasmall nanoparticles, interactions with proteins may modulate cellular uptake. Establishing highly defined model systems for such ultrasmall nanoparticles remains particularly challenging, since conventional analytical procedures developed for ordinary nanomaterials are often inadequate to resolve weakly bound protein species and dynamic corona exchange. The combination of fluorescence spectroscopy, chromatography, and confocal microscopy enabled us to qualitatively characterize these systems and infer their main interaction trends. Such mechanistic insight is crucial for the rational design of nanocarriers with tailored corona composition, paving the way for improved targeting efficiency and safer biomedical applications.^21,23,46^

## Materials and Methods

### CD Synthesis

Microwave-assisted synthesis of fluorescent N-doped carbon nanodots (CDs) using arginine and ethylenediamine as carbon and nitrogen precursors was carried out following a previously reported procedure (Figure S1).^24,25^ Characterization according to literature procedures can be found in the Supporting Information (SI): nanoparticle morphology as determined by atomic force microscopy (AFM, Figure S2a) and transmission electron microscopy (TEM, Figure S5a); colloidal properties as probed by dynamic light scattering (DLS, Figure S5d); and light absorption, fluorescence emission, and fluorescence quantum yield (FLQY = 0.17; Figure S3a,c).^24^

### Preparation of CD-HSA Complexes

Human serum albumin (HSA) was fluorescence-labeled with cyanine dye (HSA-Cy) according to the manufacturer’s instructions (Lumiprobe, Supporting Information – Labeling of HSA with Cy-7). The CDs were preincubated with HSA or HSA-Cy, resulting in the formation of preformed CD-HSA ensembles, which were purified by size exclusion chromatography (SEC, PD-10 desalting column from Cytiva #17085101, exclusion limit ca. 5 kDa) and kept at 4 °C until use. Elution was performed by gravity flow according to the manufacturer’s instructions, collecting 1 mL fractions sequentially. Calibration with control samples showed that free HSA eluted in fractions 2–3 (Figure S7), while CDs eluted in fractions 3–16 due to partial retention within the gel pores (Figure S6). Mixed samples containing preincubated CDs-HSA displayed intermediate elution behavior, confirming the formation of assemblies. The partial overlap observed for CDs-HSA mixtures suggests weak, reversible adsorption, consistent with the dynamic corona behavior previously described for ultrasmall nanoparticles.^34,35^ Although the interaction between CDs and HSA is dynamic and reversible, the consistent optical and cellular responses observed across experiments suggest that the system reaches a stable equilibrium during incubation, maintaining a representative preformed corona under the applied conditions.^34^

### Characterization Techniques

The size of CDs and CDs-HSA-Cy was characterized by atomic force microscopy (AFM, Figure S5a,c), transmission electron microscopy (TEM, Figure S5b), and dynamic light scattering (DLS, Figure S5d). AFM images were obtained with a Nanoscope IIIa, VEECO Instruments, working in tapping mode with an HQ:NSC19/ALBS probe (80 kHz; 0.6 N/m) (MikroMasch). AFM images were analyzed in Gwyddion 2.41. TEM images were acquired with a JEOL JEM 2100F microscope. For imaging, CD samples were prepared on glow-discharged carbon film 400 copper mesh grids from Electron Microscopy Sciences (Hatfield, USA), and uranyl acetate purchased from Electron Microscopy Science was used for the negative staining^48^ of the samples. ImageJ software was used for the analysis of the images. A Malvern Zetasizer, equipped with a 10 mW He–Ne laser operating at a wavelength of 633 nm, was used to measure the hydrodynamic diameter d_h_ (with dynamic light scattering, DLS) and the zeta-potential ζ (with laser Doppler anemometry, LDA) of the samples.^35^ UV–vis absorption spectra were recorded on a Jasco V-630 Bio spectrophotometer Jasco Analytical Instruments. Fluorescence spectra were measured on an LS55 PerkinElmer fluorimeter. All of the spectra were recorded at room temperature using 10 mm path-length cuvettes. The relative quantum yield (QY) Φ of the as-synthesized CDs was determined using quinine sulfate in 0.1 M H_2_SO_4_ (Φ = 54%) as reference standard with a protocol reported in the literature.^49^

### Flow Cytometry

125,000 HeLa cells in 1 mL of 10% FBS-supplemented DMEM (Dulbecco’s Modified Eagle Medium) were seeded in 12-well plates (#150628, Nunc, growth area per well: 3.5 cm^2^) and incubated overnight. On the next day, the medium was removed, and cells were incubated in fresh either serum-free or 10% FBS-supplemented medium under the presence of CDs or CDs-HSA-Cy (*C*_CD_ = 100, 200, 400 μg mL^–1^) for 24 or 48 h. The weight refers to the CD part of the conjugates. The CDs and HSA-Cy quantities hereby were determined by, respectively, fluorescence calibration curve and Bradford assay right after size exclusion chromatography purification (see the “Preparation of CD-HSA complexes” subsection and Table S1). Then, cells were trypsinized, collected in a tube, and analyzed by flow cytometry using a 3-laser BD FACS Canto II system, and data analysis was performed with FACSDiva software. For the measurements, 10,000 cells were analyzed by the flow cytometer, collecting signals from two fluorescence channels: the blue channel (laser line for excitation: 405 nm, band-pass filter: 450 nm/50 nm) for measuring the fluorescence signal from the CDs, and the red channel (laser line for excitation: 633 nm, band-pass filter: 660 nm/20 nm) for collecting the fluorescence from the labeled protein HSA-Cy. Prior to any sample analysis, the manufacturer’s quality control (QC) checks and calibration using CS&T beads (BD Biosciences) were performed to evaluate system performance and adjust the settings against baseline values. For the cells exposed to CDs, experiments were repeated three times. For the cells exposed to CDs-HSA-Cy, the experiments were repeated twice.

### Confocal Fluorescence Microscopy

35,000 HeLa cells in 300 μL of 10% FBS-supplemented DMEM medium were seeded in 8-well chambered cover glass (no. 80806, ibidi, growth area per well: 1.0 cm^2^) and incubated overnight. On the next day, the medium was removed, and cells were incubated in fresh medium (either serum-free or 10% FBS-supplemented) with CDs, CD-HSA, or CDs-HSA-Cy (*C*_CD_ = 100, 200, 400 μg mL^–1^) for 6, 24, or 48 h. Cells were gently washed with phosphate-buffered saline (PBS) three times. For cell imaging, a confocal laser scanning microscope (CLSM 510 Meta, Zeiss) with a Plan-Apochromat 63*x*/1.40 Oil DIC M27 objective was used. Frame scanning with the 405 and 633 nm lasers was performed in sequential channel mode for collecting the emission of CDs and HSA-Cy at 420–500 and 650–750 nm, respectively. The transmission field was collected by using a 633 nm laser. Images were scanned in the sequential channel mode.

## Supplementary Material



## Notes

Labeling of HSA with Cy-7 (HSA-Cy), plots of CD concentration CCD versus the fluorescence emission intensity I_CD_, absorption and fluorescence emission characterization, UV-vis absorption spectrum, size characterization of CDs, three times repeated elution tests, CLSM images of HeLa cells, concentration-dependent uptake study, summarized results of detected CDs and HSA, and calculated concentrations of CDs and HSA (PDF)

## ◆

Hangzhou Normal University, Key Laboratory of Organosilicon Chemistry and Material Technology, Ministry of Education, Zhejiang Key Laboratory of Organosilicon Material Technology, College of Material, Chemistry and Chemical Engineering, Hangzhou, Zhejiang 311121, China
