# Supplementary material for: Impact of Proteins on the Cellular Uptake of Carbon Nanodots
Source: ACS Omega. 2025 Dec 3;10(49):60928–35. doi: 10.1021/acsomega.5c09510 (PMC12713424; doi:10.1021/acsomega.5c09510)
Supplement: Supplementary file 1 [file ao5c09510_si_001.pdf]

Impact of proteins on the cellular uptake of carbon nanodots

Ziyao Liu,[1,‡] Huijie Yan,[1,‡] I. Jénnifer Gómez,[2,3] Carolina Carrillo Carion,[2,4] Blanca Arnaiz,[2] Dingcheng Zhu,[1] Maurizio Prato,[2,5,6] Wolfgang J. Parak,[1] Neus Feliu,[1,*] Michele Cacioppo[1,2,5,7]*

[1] *Universität Hamburg Fachbereich Physik, ChyN, Luruper Chaussee 149, Hamburg, DE 22607*
[2]*Center for Cooperative Research in Biomaterials (CIC biomaGUNE), Basque Research and Technology Alliance (BRTA), San Sebastián, PV, ES 20014*
[3]*Universidade da Coruña, Centro Interdisciplinar de Química e Bioloxía (CICA), Rúa as Carballeiras, A Coruna, ES 15001*
[4]*Institute for Chemical Research (IIQ), CSIC-University of Sevilla, Sevilla, ES 41092*
[5] *Dipartimento di Scienze Chimiche e Farmaceutiche (DSCF), Via L. Giorgieri 1, Università degli Studi di Trieste, Trieste, IT 34127*
[6] *Basque Foundation for Science, Ikerbasque, Bilbao, PV, ES 48013*
[7] *Dipartimento di Scienze e Tecnologie Biologiche Chimiche e Farmaceutiche (STEBICEF), Viale delle Scienze – Ed. 17, Università degli Studi di Palermo, Palermo, IT 90128*

[*]*Corresponding authors: neus.feliu@physnet.uni-hamburg.de, michele.cacioppo@unipa.it*
[‡]*These authors equally contributed to the work*

SUPPORTING INFORMATION

Supporting Procedures

Labeling of HSA with Cy7 (HSA-Cy)

Human serum albumin (HSA) was labeled with the fluorescent dye sulfo-cyanine7 NHS ester (Cy7, maximum absorption/emission wavelengths $\lambda_{abs}/\lambda_{em}$ = 750 nm / 773 nm, molar extinction coefficient ε_{Cy7}(750 nm) = 2.40 x 10^5 $M^{-1}\cdot cm^{-1}$). The labeling procedure was carried out following the protocol recommended by the supplier (Lumiprobe, Sulfo-Cyanin7 NHS-Ester, cat: #25320). The dye dissolved in dimethylformamide (DMF) at 10 $mg\cdot mL^{-1}$ was added to the HSA protein previously dissolved in sodium bicarbonate buffer (100 mM, pH = 8.50) at 10 $mg\cdot mL^{-1}$, and the mixture was incubated at 25 °C under gentle stirring for 5 h. After that, unreacted Cy7 dye was separated from labeled HSA by size exclusion chromatography on a PD-10 column using HEPES buffer (100 mM, pH = 8.5) for the elution of the HSA-Cy fraction. This fraction was then washed with HEPES buffer by using AMICON filters (30 kDa cut-off) to remove remaining free Cy7 molecules, until no free Cy7 was found in the filtrates. Finally, the purified HSA-Cy solution was stored at 4 °C until use. By UV-vis absorption spectroscopy, that is the average number of fluorophore molecules linked to a protein molecule, was determined to be *ca.* 1.20.

The Φ_{DOL} value and the molar concentration (c_{HSA} (M)) and mass concentration (C_{HSA} ($mg\cdot mL^{-1}$)) of the conjugates were calculated according to the following equations, following the protol of the supplier (https://www.lumiprobe.com/protocols/nhs-ester-labeling):

$$\Phi_{DOL} = \frac{(A(750\,nm)\cdot\varepsilon_{HSA}(280\,nm))}{(A(280\,nm) - A(750\,nm)\cdot\eta_{CF})\cdot\varepsilon_{Cy7}(750\,nm))} \quad \text{S1}$$

$$c_{HSA-Cy} = \frac{(A(280\,nm) - A(750\,nm)\cdot\eta_{CF})}{\varepsilon_{HSA}(280\,nm)} \quad \text{S2}$$

$$C_{HSA-Cy} \approx c_{HSA-Cy}\cdot M_{HSA} \quad \text{S3}$$

where A(280 nm) is the absorbance of the conjugate solution measured at 280 nm where predominantly HSA absorbs, A(750 nm) is the absorbance of the conjugate solution measured at λ_{exc} = 750 nM where predominantly Cy7 absorbs. ε_{Cy7}(750 nm) and η_{CF} (correction factor) are the intrinsic properties of the dye; ε_{HSA}(280 nm)[1] = 35700 $M^{-1}\cdot cm^{-1}$ and M_{HSA} = 66500 $g\cdot mol^{-1}$ are the molar extinction coefficient and the molar mass of HSA and are intrinsic properties of HSA.

The mild labeling procedure and the low dye-to-protein ratio (ca. 1.2 dyes per HSA molecule) are expected to preserve the native conformation and binding capacity of HSA. Therefore, potential alterations induced by dye conjugation are considered negligible compared with the supramolecular interaction effects studied here.[2,3]

Supporting Figures

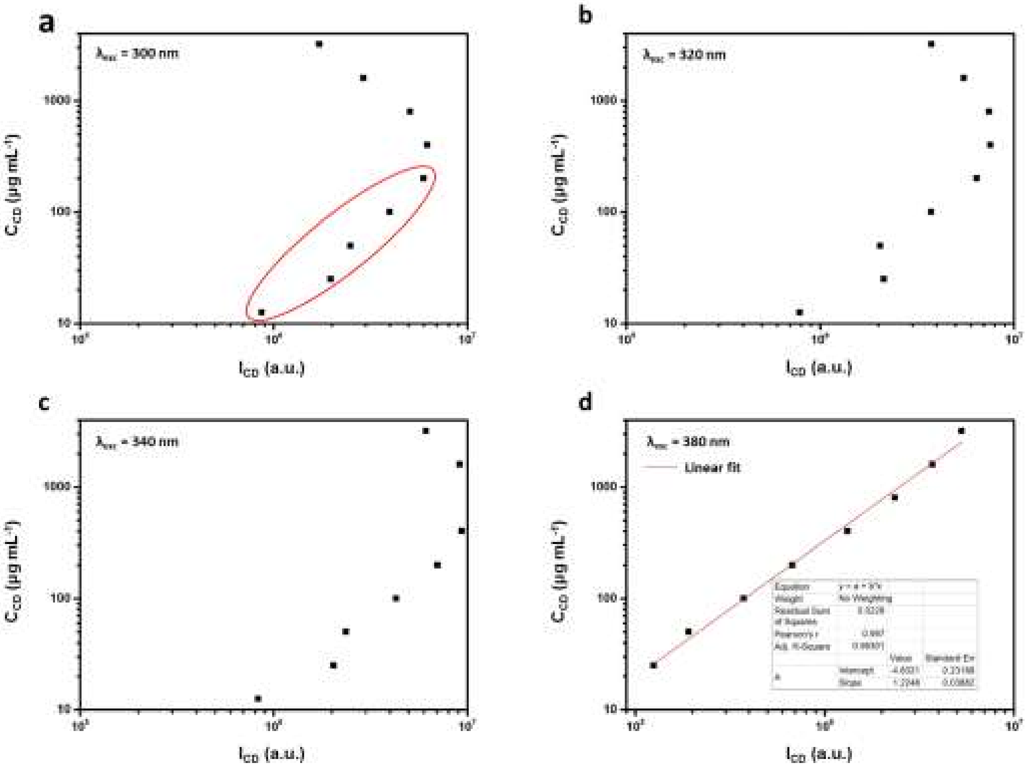

Figure S1. Plots of CD concentration C_{CD} versus the fluorescence emission intensity I_{CD} of a CD solution under different excitation wavelengths a) λ_{exc} = 300 nm, λ_{em} = 310 nm, b) λ_{exc} = 320 nm, λ_{em} = 330 nm, c) λ_{exc} = 340 nm, λ_{em} = 350 nm, and d) λ_{exc} = 380 nm, λ_{em} = 390 nm, as measured with a fluorescence spectrometer (Fluorolog-3, Horiba Jobin Yvon, USA) by diluting a stock solution (5 mg mL^{-1}) in PBS medium . In (d) the double logarithmic curve has been fitted by linear regression analysis with $\log(C_{CD}/(\mu g \cdot mL^{-1}))$ = -4.8 + 1.2 $\cdot\log(I_{CD})$ (coefficient of determination R^2 = 0.99301).

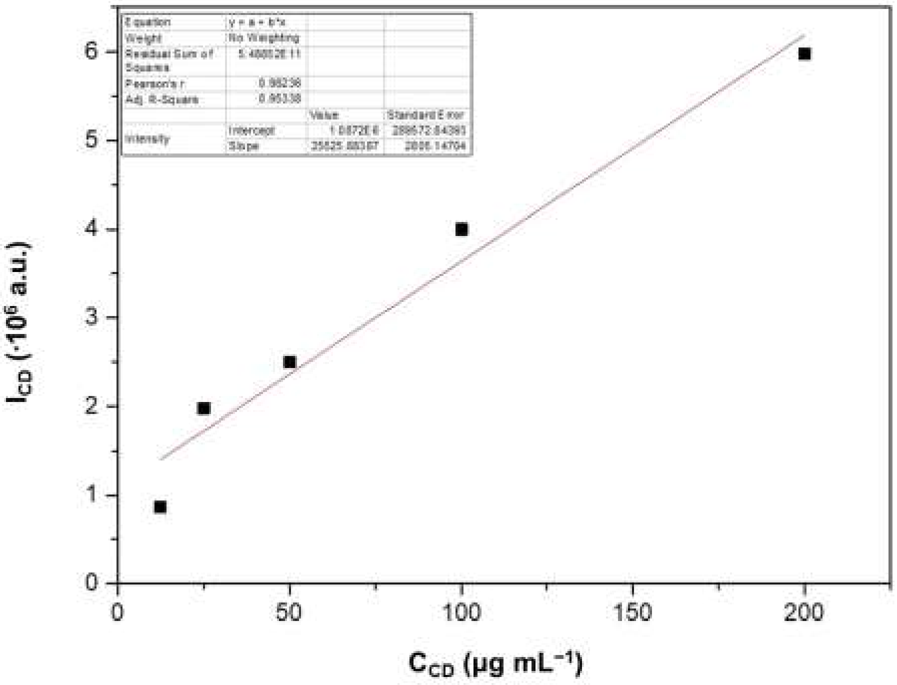

Figure S2. Plot of CD fluorescence emission intensity I_{CD}, under excitation wavelength 300 nm, versus the CD concentration C_{CD} in a solution of CDs dispersed in PBS, as measured with a fluorescence spectrometer (Fluorolog-3, Horiba Jobin Yvon, USA) by diluting a stock solution (5 mg·mL^{-1}). The data points shown here correspond to the data from Figure S1 which are inside the red boarder line. The data were fitted by linear regression analysis with I_{CD} = (1.09·10^6 + 2.55·10^5 mL·μg^{-1} · C_{CD}).

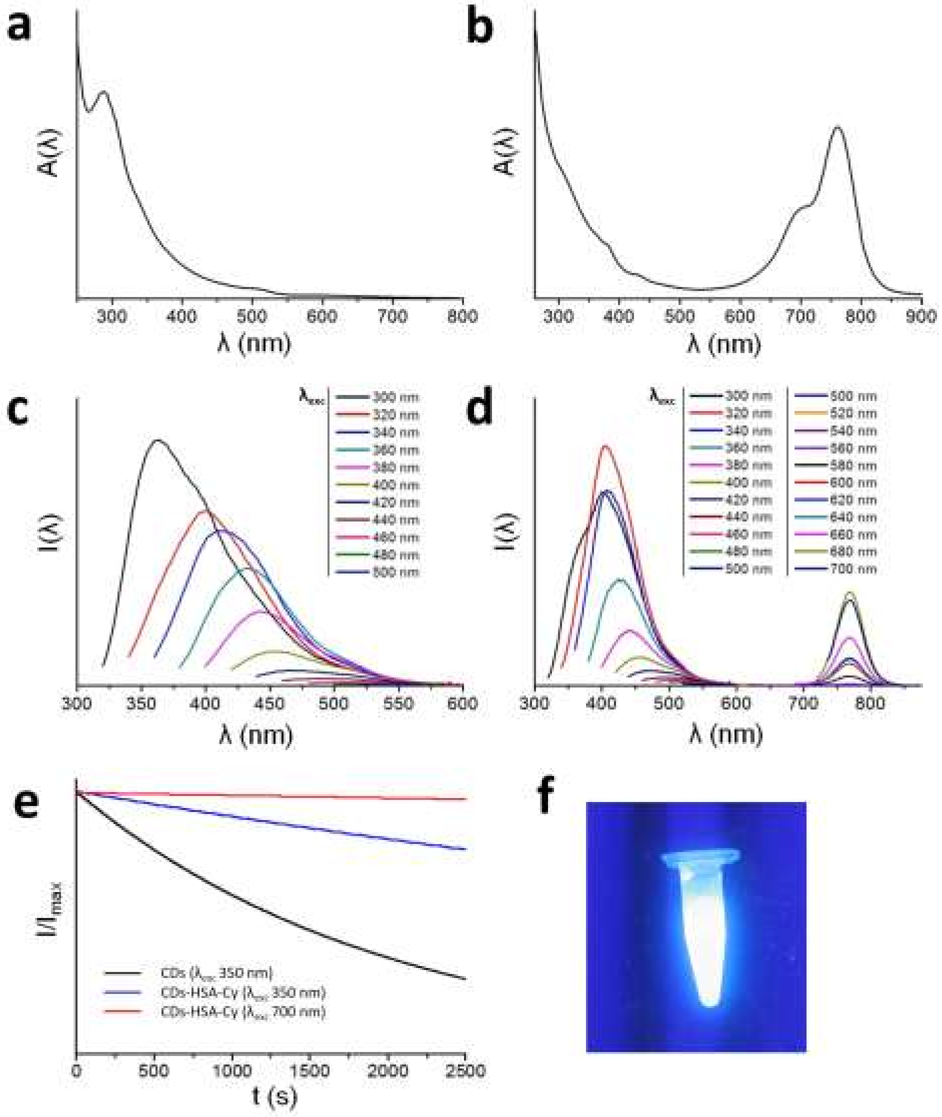

Figure S3. Absorption (a,b) and fluorescence (c,d) emission characterization of naked CDs and CDs-HSA-Cy as recorded in PBS. UV-vis absorption spectra of CDs (a) and CDs-HSA-Cy (b). Fluorescence emission spectra of CDs (c) and CDs-HSA-Cy (d) at different excitation wavelengths λ_{exc}. Photostability of the fluorescence emission of CDs, and both emissions (i.e. CD and dye) from CDs-HSA-Cy, under continuous irradiation at 350 nm or 700 nm for the time t (e). Photograph of an Eppendorf tube containing a 1 mg·mL^{-1} aqueous solution of CDs under UV lamp irradiation (f).

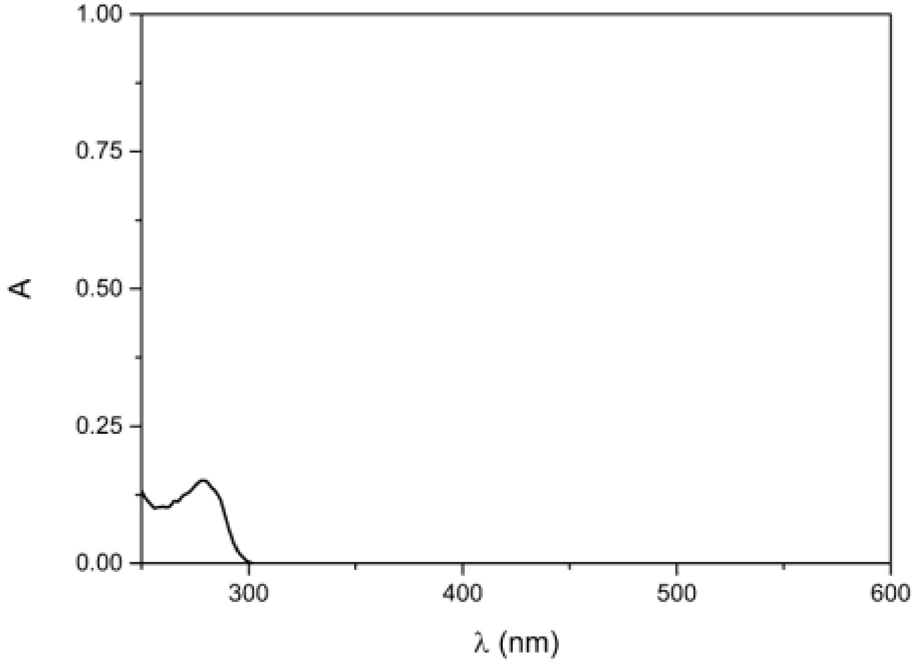

Figure S4. UV-vis absorption spectrum A(λ) of a C_{HSA} = 300 µg·mL^{-1} solution of HSA in phosphate buffered saline (PBS) buffer.

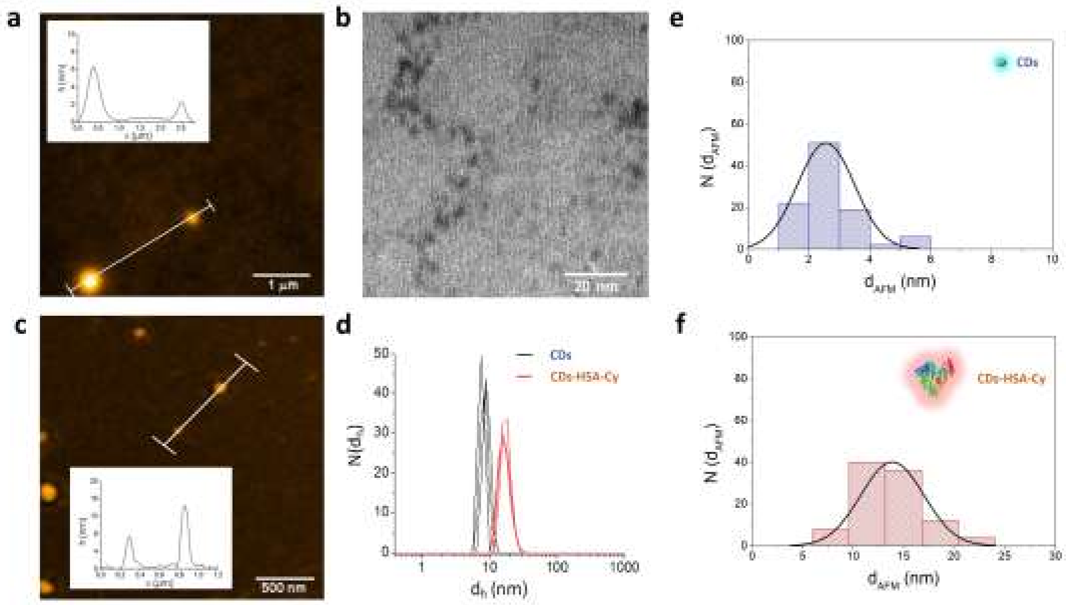

Figure S5. Size characterization of CDs and modified with an artificial fluorescence-labeled protein corona (CDs-HSA-Cy). (a) Tapping mode AFM topography image of CDs on a mica substrate, and the corresponding height-profile h(x) analysis along the line in x-direction. Note

that the height of a nanoparticle in an AFM image is related to its diameter; (b) Negative staining TEM image of CDs; (c) AFM topography image of CDs-HSA-Cy on a mica substrate, and the corresponding height-profile analysis along the line; (d) Hydrodynamic diameter d_h given as number distribution $N(d_h)$ as determined by DLS; (e) Height distribution histogram of the CDs $N(d_{AFM})$, based on AFM data with over imposed distribution curve (black line), from which from the height profile the diameter d_{AFM} of the CDs has been evaluated; (f) CDs-HSA-Cy height distribution histogram $N(d_{AFM})$ based on AFM data with over imposed distribution curve (black line).

Fractions

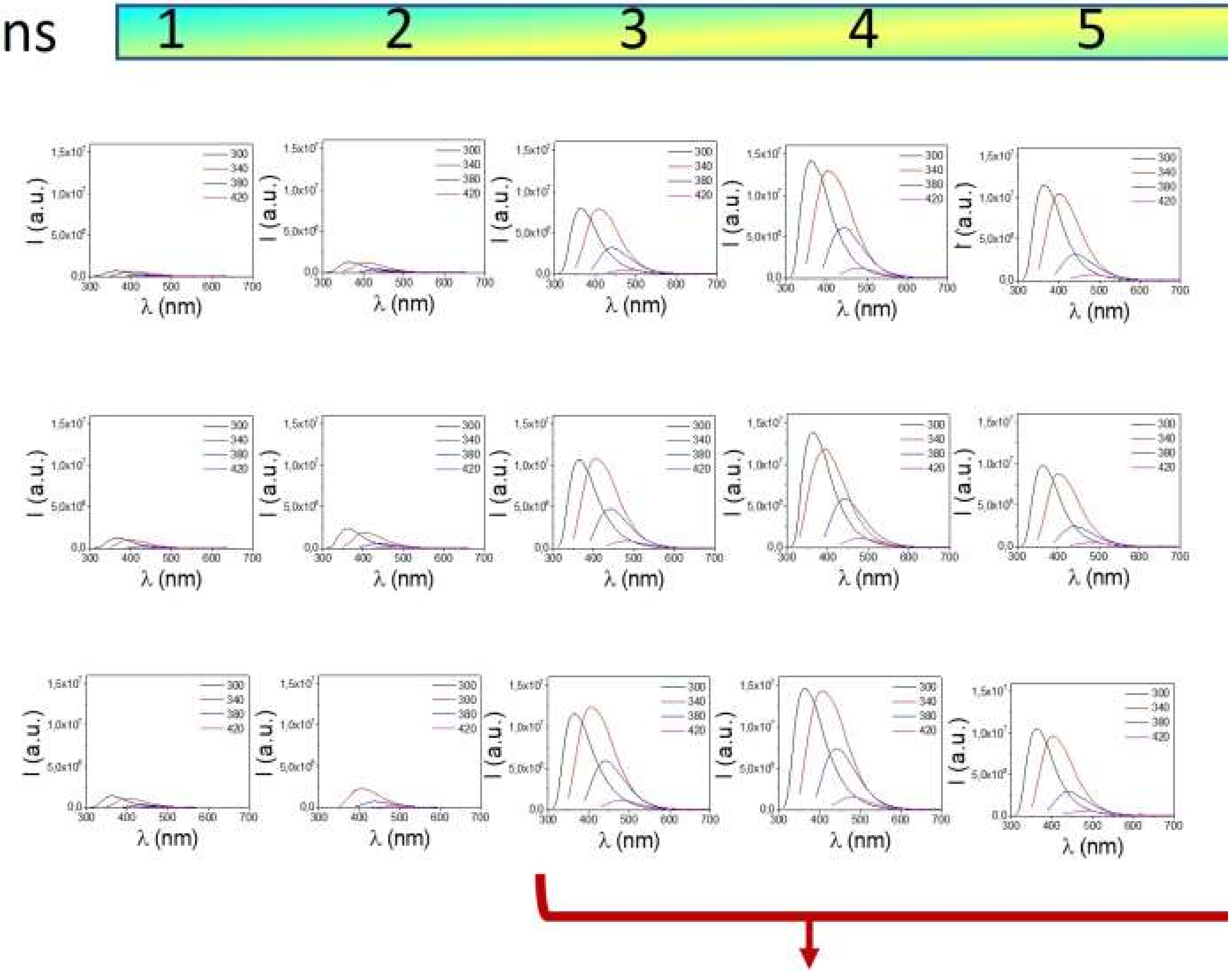

1st column

2nd column

3rd column

High concentration fraction

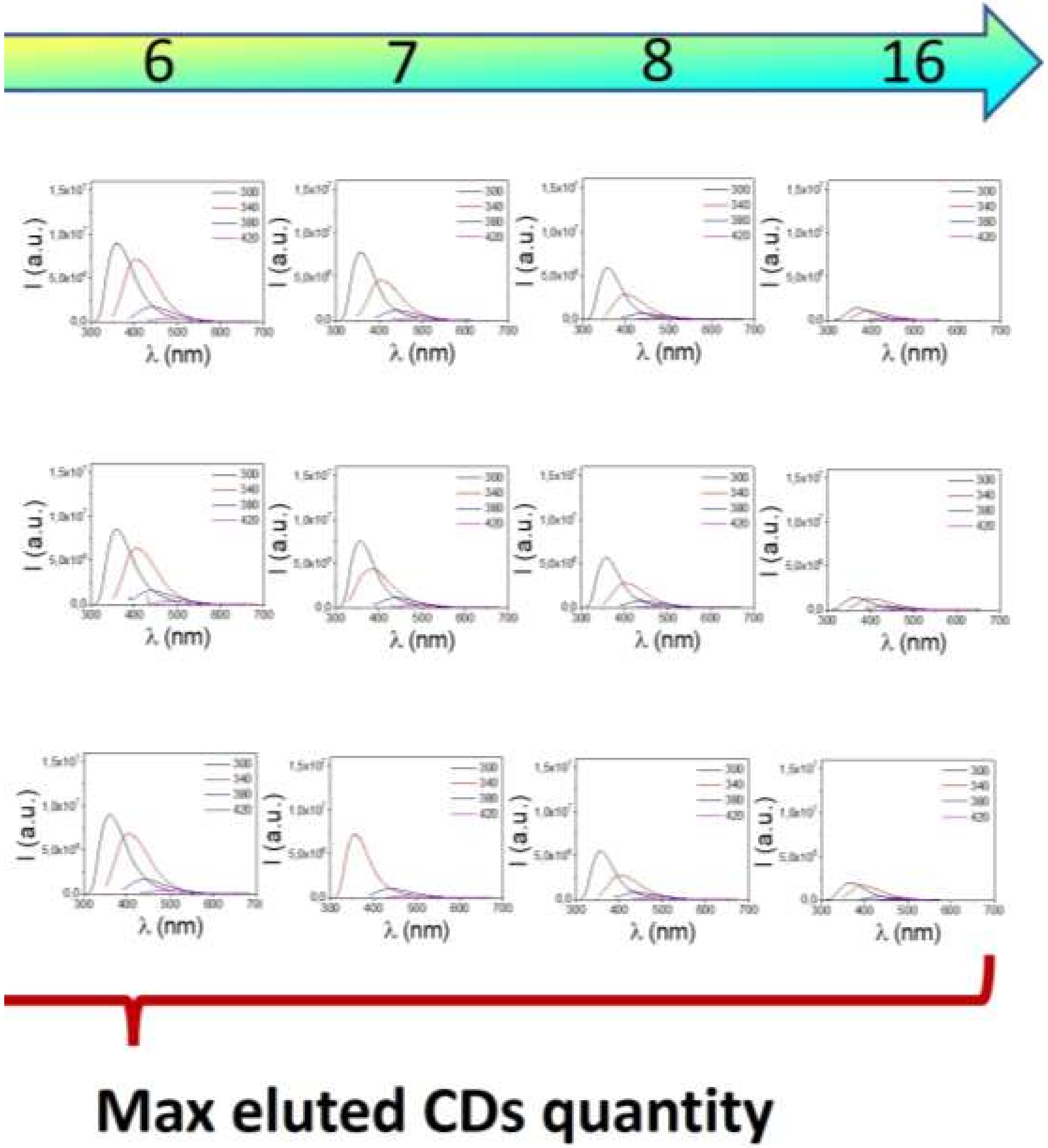

Figure S6. Three times repeated elution experiments of CD solution (m_{CD} = 1 mg, C_{CD} = 1000 µg·mL^{-1}, V = 1 mL; $m_{CD} = C_{CD}·V$) in 22,5 mL PD-10 columns using PBS buffer as

eluent. The CDs in the eluted fractions (1 mL each) were detected using fluorescence emission spectroscopy I(λ) exciting at λ_{exc} = 300 nm (black line), 340 nm (red line), 380 nm (blue line), and 420 nm (purple line). The highest content of CDs was found in fractions #3 - #16. The four excitation wavelengths (300, 340, 380, 420 nm) were used to confirm the excitation-dependent fluorescence of CDs. The spectra demonstrate the consistent emission profile of CDs under varying excitation conditions. The graphs are enlarged to fit two pages for better readability.

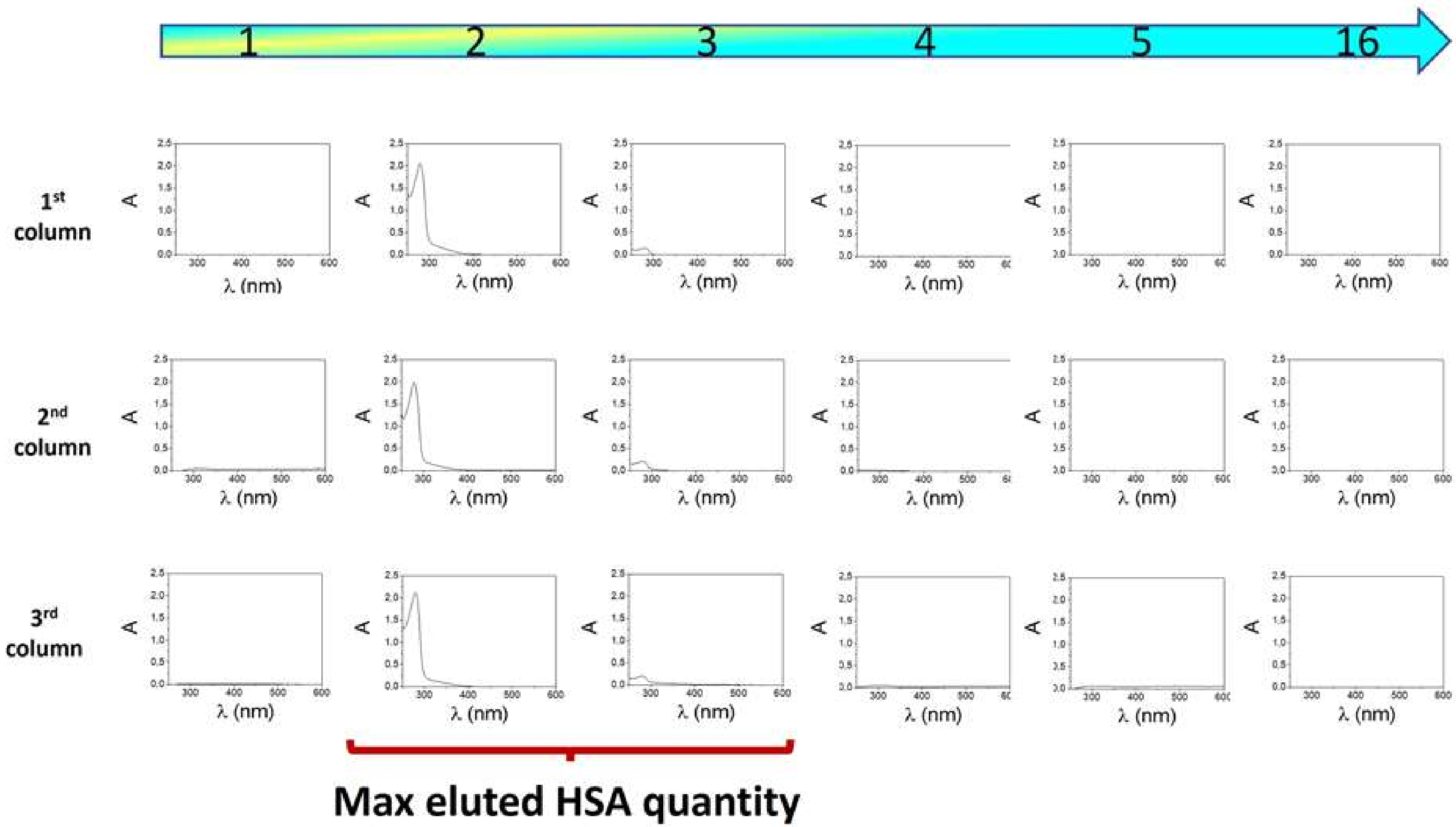

Figure S7. Three times repeated elution tests of HSA (m_{HSA} = 10 mg; C_{HSA} = 10000 µg·mL^{-1}; V = 1 mL; $m_{HSA} = C_{HSA}$·V) in 22,5 mL PD-10 columns using PBS buffer as eluent. The HSA content in the eluted fractions (1 mL each) was detected using UV-vis absorbance spectroscopy A(λ). The highest content of HSA was found in fractions #2 - #3.

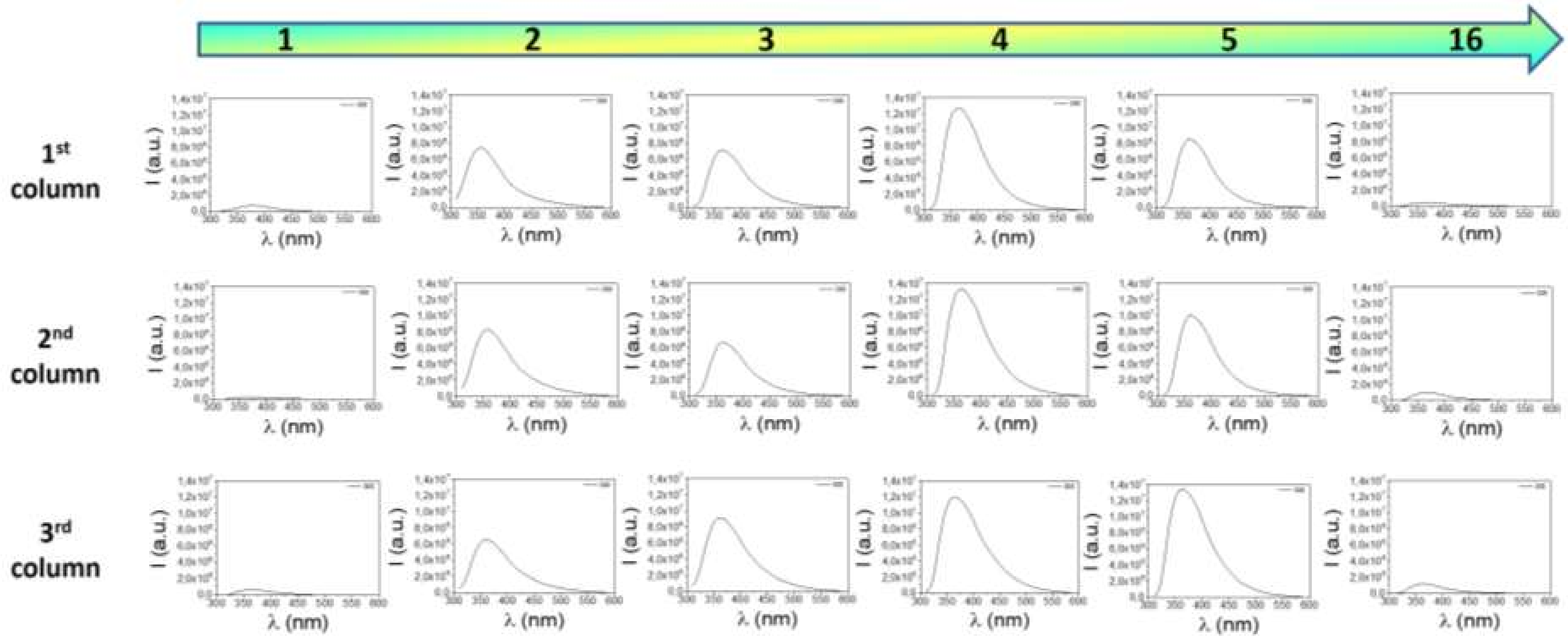

Figure S8. Three times repeated elution tests of CDs-HSA (1:10 w/w; CDs were incubated with HSA for 1 hour to form pre-coated CDs) in 22,5 mL PD-10 columns using PBS buffer as eluent. The content of nanoparticles (may comprise CDs-HSA, CDs, and HSA) in the eluted fractions (1 mL each) were detected using fluorescence spectroscopy I(λ) (this graph) and the Bradford assay for HSA quantification (see Table S1).

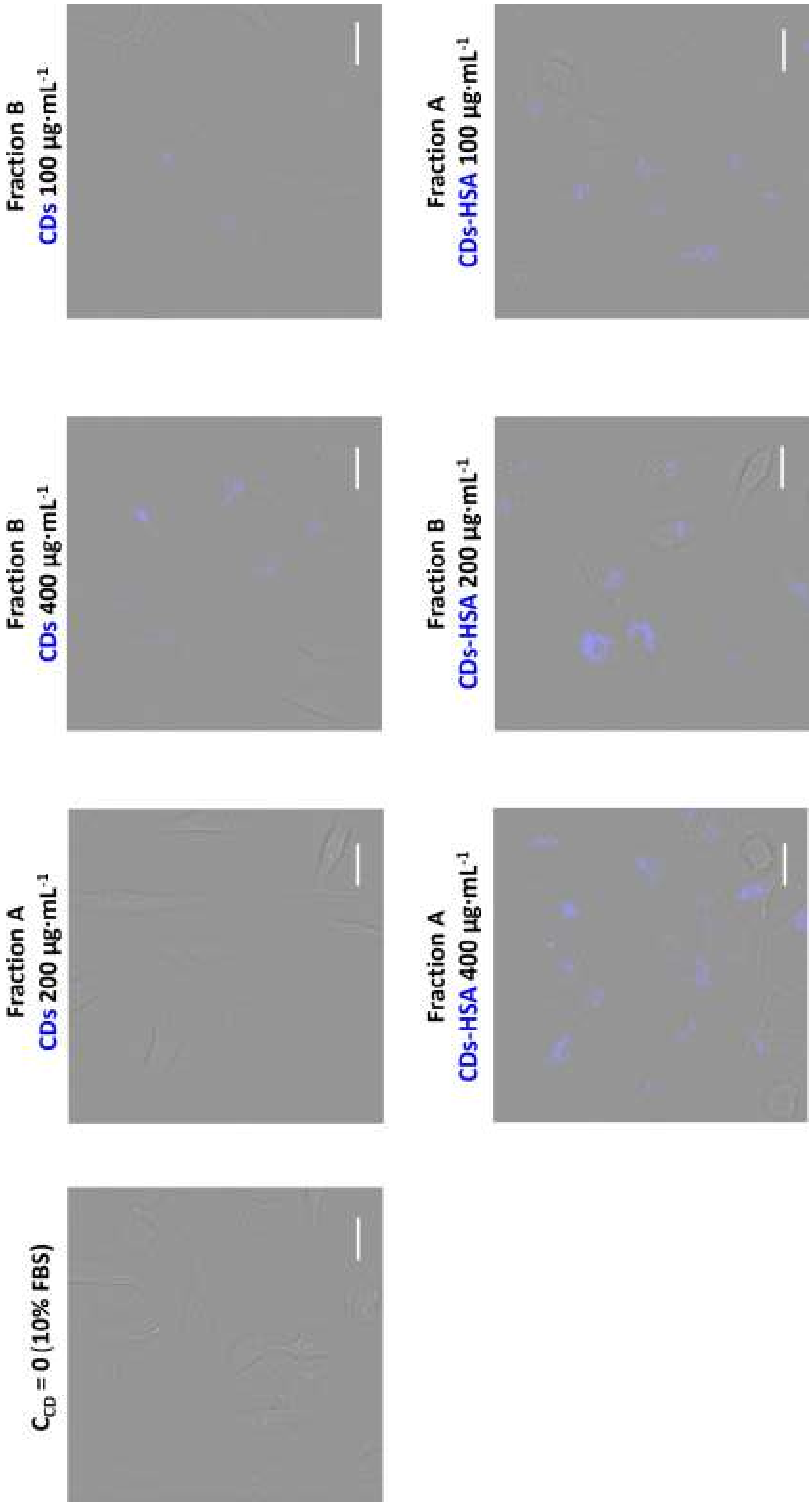

Figure S9. Confocal laser scanning microscopy (CLSM) images of HeLa cells which had been incubated for 6 h with CDs and CDs-HSA in the presence of 10% FBS supplemented medium. Scale bar: 20 μm. The laser excitation wavelength and experimental details are available in experimental section of the main article.

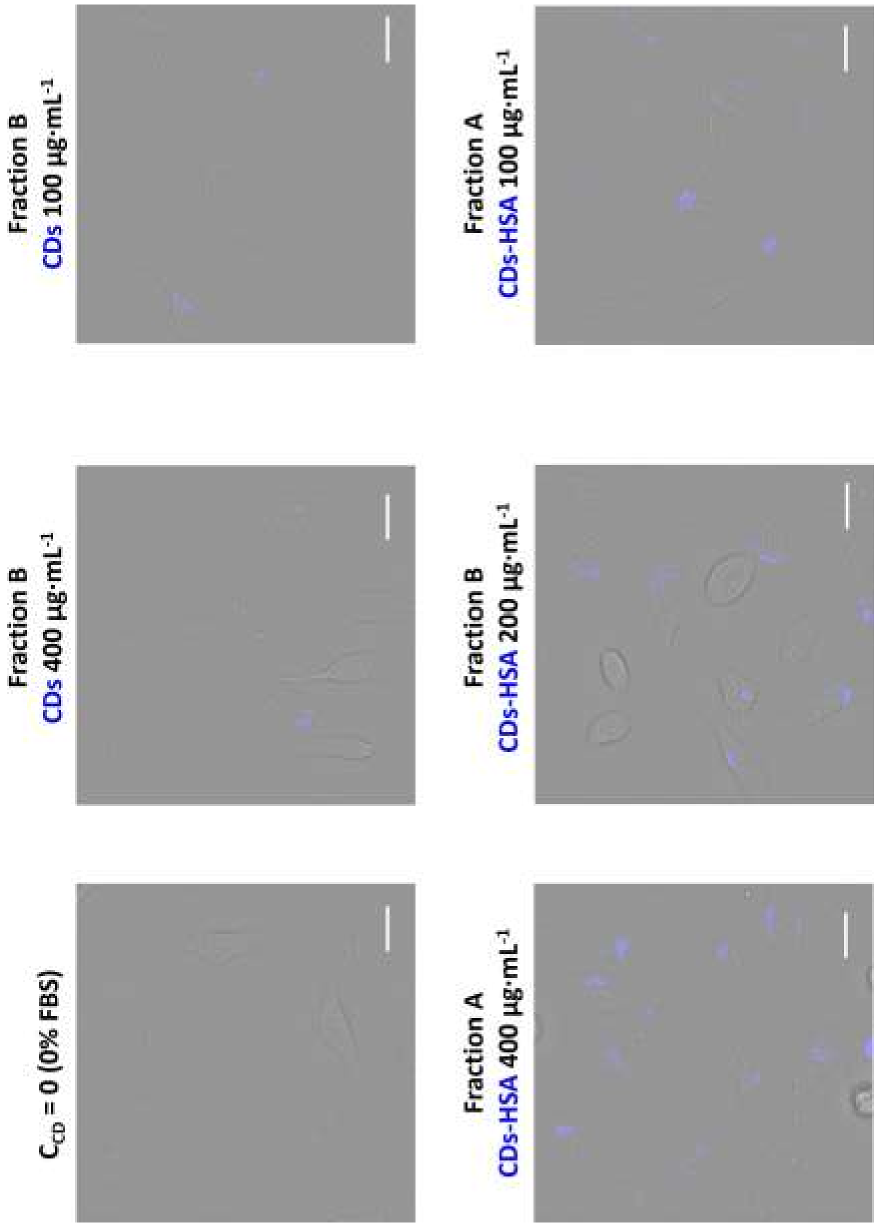

Figure S10. CLSM images of HeLa cells which had been incubated for 6 h with CDs and CDs-HSA in serum-free medium. Scale bar: 20 µm. The laser excitation wavelength and experimental details are available in Experimental section of the main text.

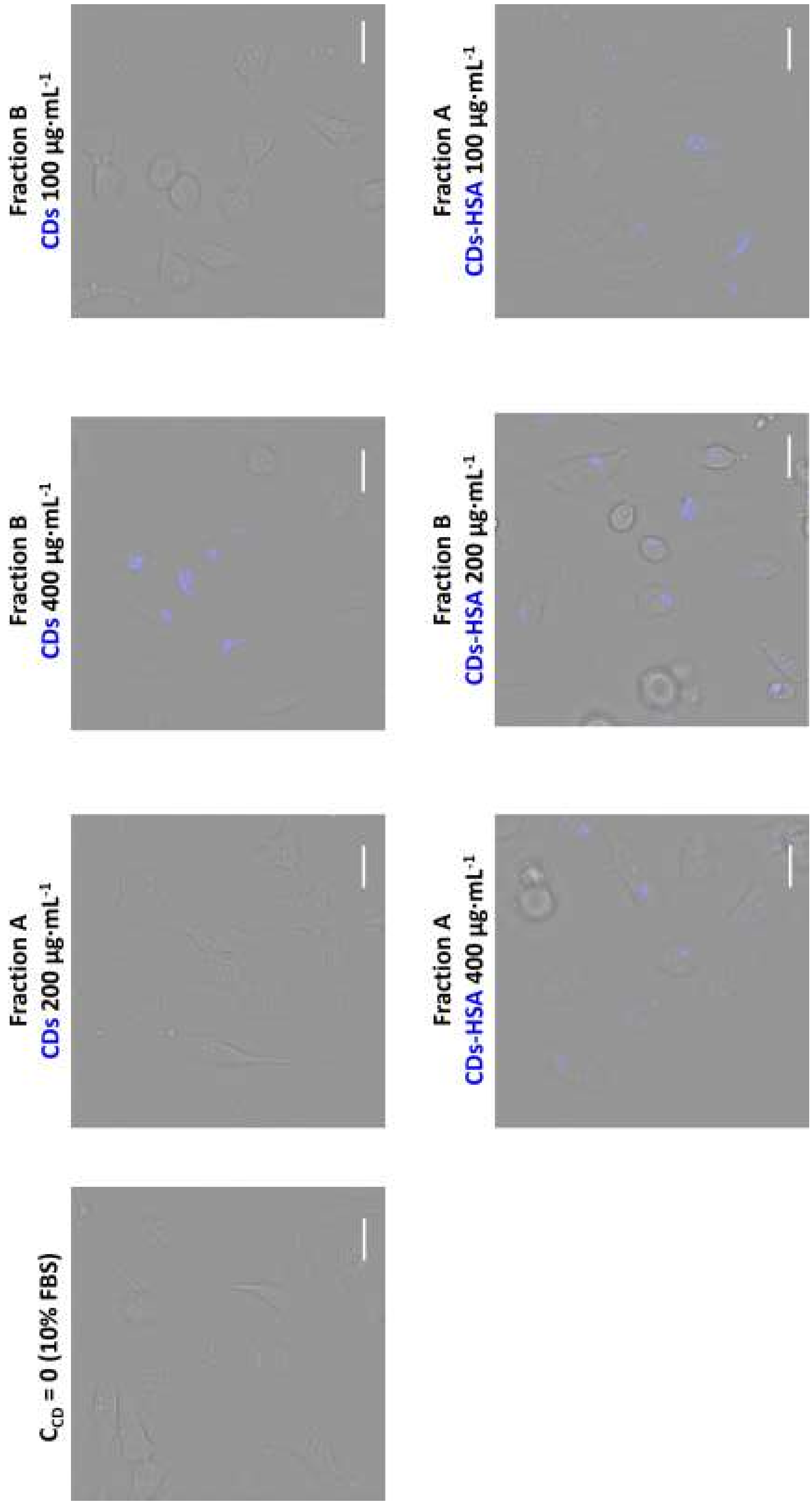

Figure S11. CLSM images of HeLa cells which had been incubated for 24 h with CDs and CDs-HSA in serum-supplemented medium. Scale bar: 20 μm. The laser excitation wavelength and experimental details are available in Experimental section of the main text.

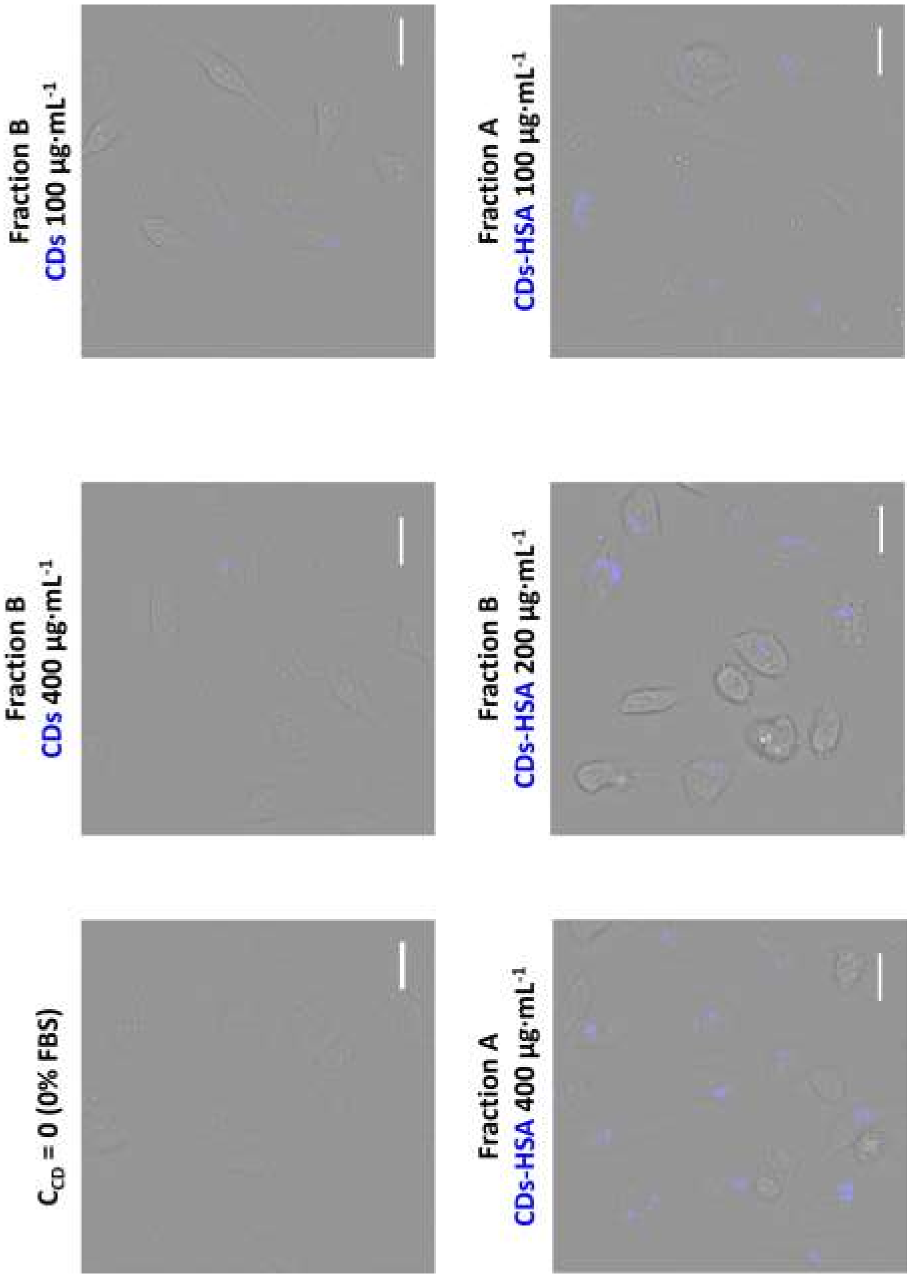

Figure S12. CLSM images of HeLa cells which had been incubated for 24 h with CDs and CDs-HSA in serum-free medium. Scale bar: 20 µm. The laser excitation wavelength and experimental details are available in Experimental section of the main text.

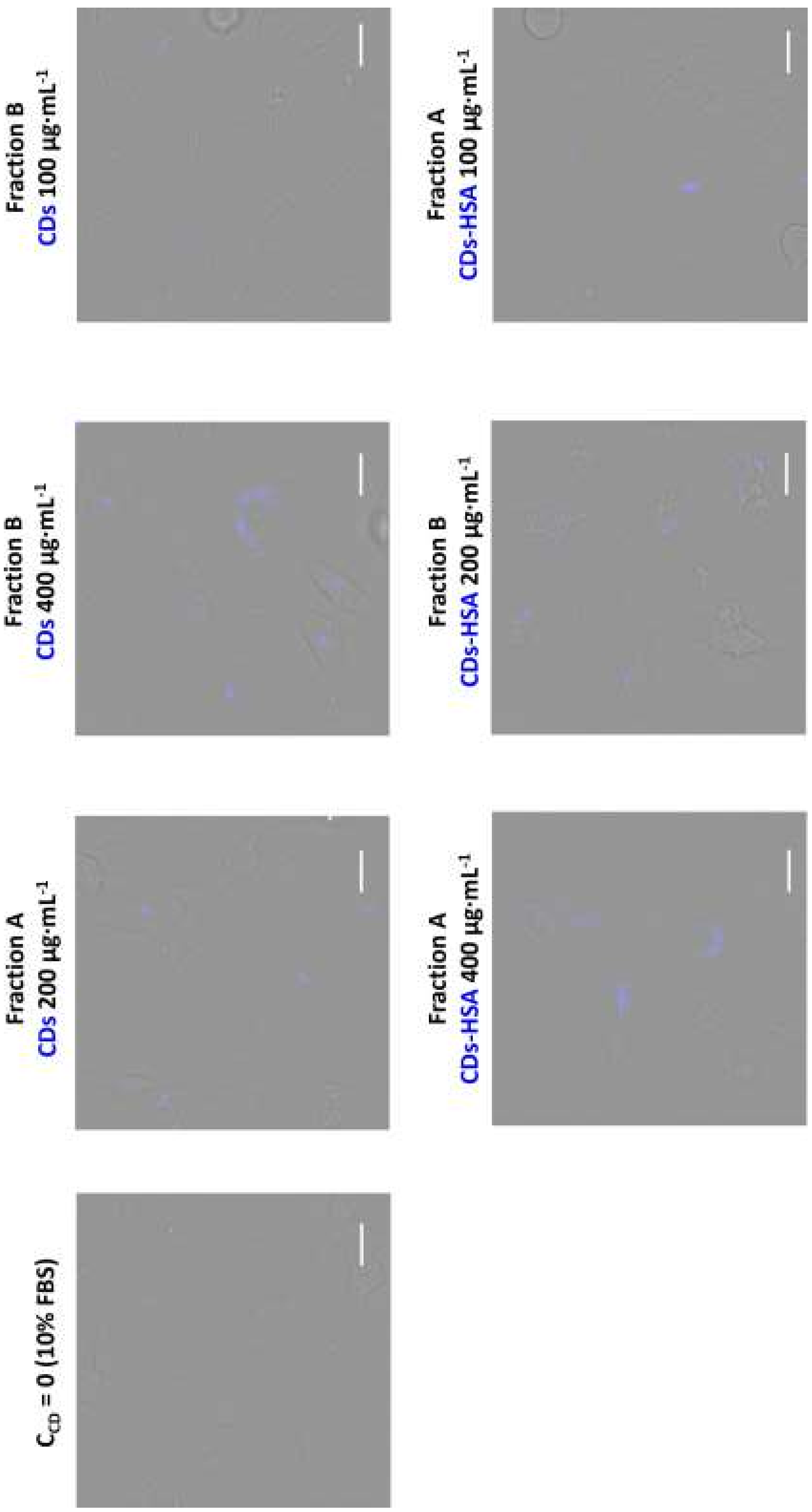

Figure S13. CLSM images of HeLa cells which had been incubated for 48 h with CDs and CDs-HSA in serum-supplemented medium. Scale bar: 20 µm. The laser excitation wavelength and experimental details are available in Experimental section of the main text.

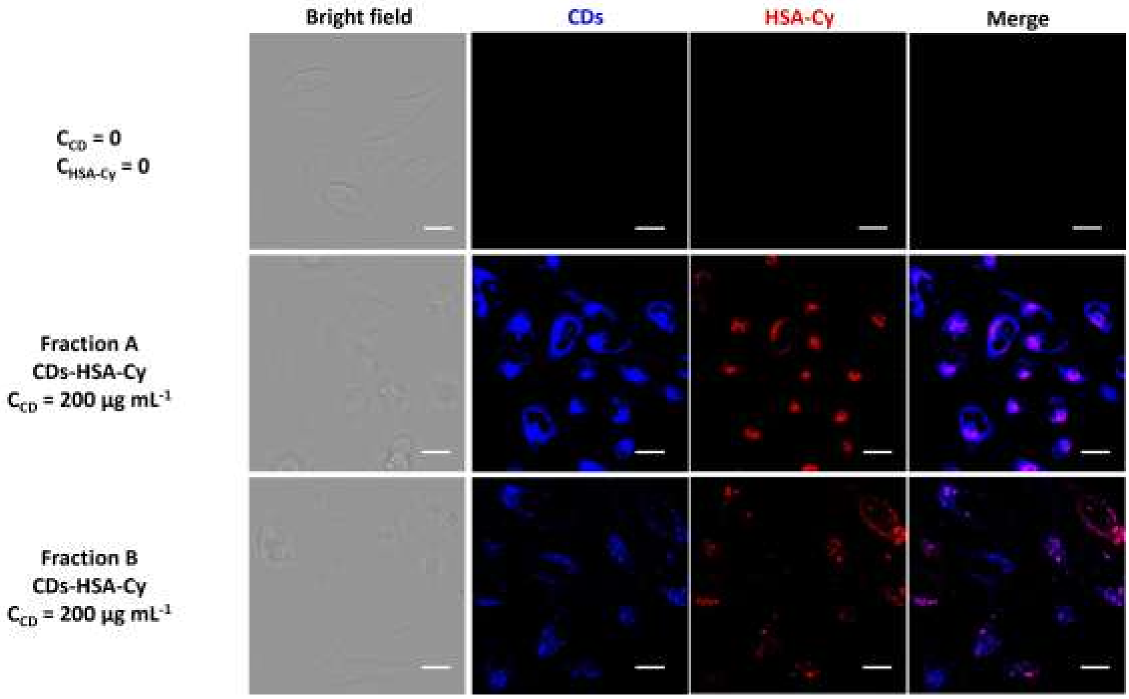

Figure S14. CLSM images of HeLa cells which had been incubated for 24 h with CDs and CDs-HSA-Cy in serum-supplemented medium. Scale bar: 20 µm. To detect the CDs and the HSA-Cy laser excitation wavelengths of λ_{exc} = 405 nm and λ_{exc} = 641 nm were used, respectively. Note, that there is fluorescence saturation in some of the images, i.e. overexposure of fluorescence at some pixels, leading to saturated intensities.

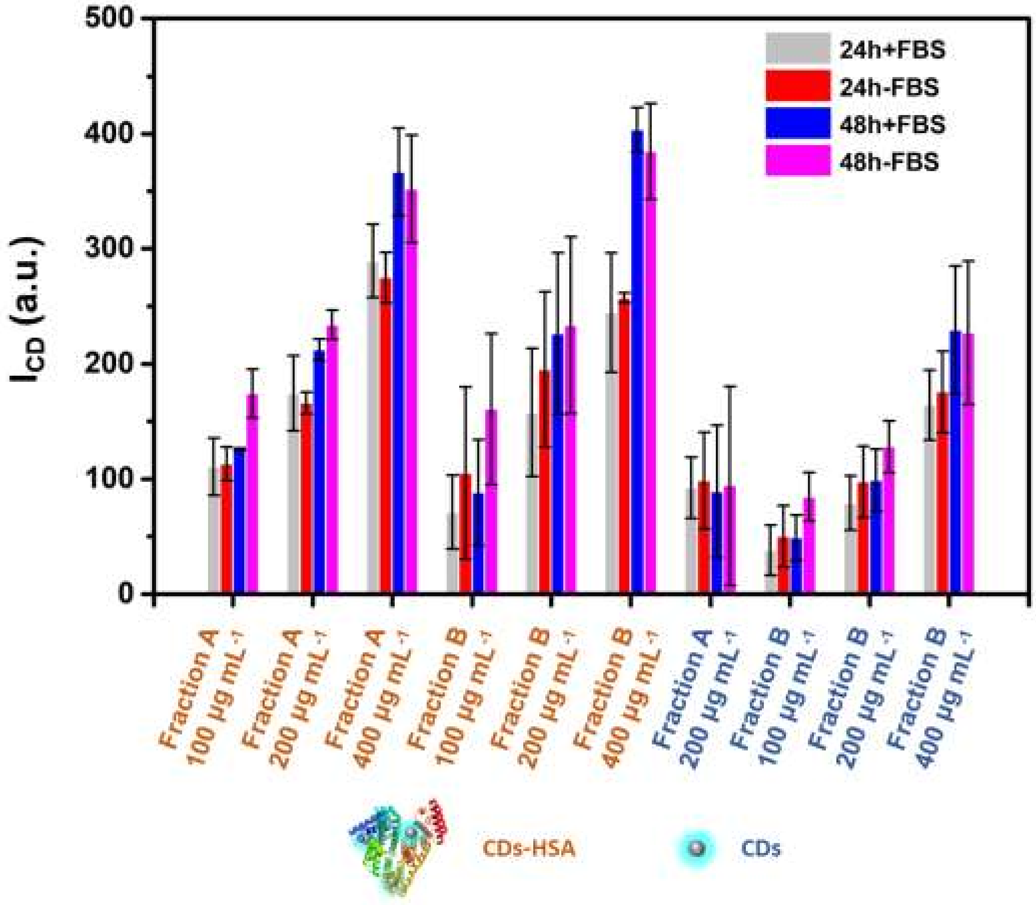

Figure S15. Concentration dependent uptake study of CDs-HSA assemblies (CD/HSA pre-incubation mass ratio 1:100) and CDs after purification by size exclusions chromatography (leading to fraction A and fraction B) by HeLa cells using confocal fluorescence microscopy. I_{CD} refers to the CD fluorescence. This graph contains the data also shown in Figure 2 and additional data.

Supporting Tables

Collected fractions	Fraction	CDs (Figure S6)	HSA (Figure S7)	CDs-HSA (Figure S8)	
	#	I_{CD} (λ_{exc} = 300 nm) (a.u)	A (λ = 280 nm)	I_{CD} (λ_{exc} = 300 nm) (a.u)	A (λ = 595 nm)
A	**1**	$(1 \pm 4)\times 10^{6}$	0.008 ± 0.007	$(6 \pm 3)\times 10^{5}$	0.53 ± 0.04
	**2**	$(2 \pm 0.6)\times 10^{6}$	2.10 ± 0.09	$(7.5 \pm 0.9)\times 10^{6}$	1.9 ± 0.3
	**3**	$(1 \pm 0.2)\times 10^{7}$	0.19 ± 0.03	$(7.7 \pm 1.0)\times 10^{6}$	1.1 ± 0.6
B	**4**	$(1.42 \pm 0.03)\times 10^{7}$	0.02 ± 0.02	$(1.27 \pm 0.06)\times 10^{7}$	0.53 ± 0.03
	**5**	$(1.06 \pm 0.09)\times 10^{7}$	0.01 ± 0.02	$(1.1 \pm 0.3)\times 10^{7}$	0.52 ± 0.03
	**16**	$(1.6 \pm 0.4)\times 10^{6}$	0 ± 0	$(9 \pm 4)\times 10^{5}$	0.47 ± 0.02

Table S1. Summarized results of detected CDs and HSA in the PD-10 elution experiments shown in Figures S6, S7 and S8. For the CDs the average emission intensity $I_{CD} = I(\lambda_{exc} = 300\text{ nm})$ was taken from Figure S6 (CDs) and from the fractions used in Figure S8 (CDs-HSA). The mass concentration C_{CD} of the CDs was then determined by using the calibration curve shown in Figure S2. The mass concentration C_{CD} was then converted into a molar concentration c_{CD} by using an assume molar mass of the CDs[4] of $M_{CD} = 1000\text{ g}\cdot\text{mol}^{-1}$: $c_{CD} = C_{CD}\cdot M_{CD}^{-1}$. The molar concentration as HSA was determined with the Bradford assay, by measuring the absorption A (λ = 595 nm) at λ = 595 nm resulting from the assay. The molar HSA concentration c_{HSA} was then calculated as + $\langle A (\lambda = 595\text{ nm})\rangle = 0.54\ \mu\text{g}\cdot\text{mL}^{-1} \cdot c_{HSA}\ 7.87\ \mu\text{g}\cdot\text{mL}^{-1}$, whereby the offset and scaling factor have been determined from a previously recorded calibration curve.

Fraction		CDs (Figure S6) c_{CD} (µmol mL^{-1})	HSA (Figure S7) c_{HSA} (µmol mL^{-1})	CDs-HSA (Figure S8)	
#				c_{CD} (µmol mL^{-1})	c_{HSA} (µmol mL^{-1})
**A**	1 - 3	0.15 ± 0.04	0.069 ± 0.007	0.51 ± 0.06	0.005 ± 0.002
**B**	4 - 16	0.85 ± 0.08	0.083 ± 0.005	0.49 ± 0.09	0.15 ± 0.03

Table S2. Calculated concentrations of CDs and HSA after collecting fractions A and B (cf. Figure S8) in 1 mL of buffer solution. For the CD-HSA conjugates the number of CDs per HSA molecule was estimated as c_{CD}/c_{HSA} and is 0.51/0.005 ≈ 100 for fraction A, and 0.49/0.15 ≈ 3 for fraction B.

References

(1) Leggio, C.; Galantini, L.; Pavel, N. V. About the Albumin Structure in Solution: Cigar Expanded Form versus Heart Normal Shape. *Physical Chemistry Chemical Physics* **2008**, *10* (45), 6741. https://doi.org/10.1039/b808938h.

(2) Usama, S. M.; Thapaliya, E. R.; Luciano, M. P.; Schnermann, M. J. Not so Innocent: Impact of Fluorophore Chemistry on the in Vivo Properties of Bioconjugates. *Curr Opin Chem Biol* **2021**, *63*, 38–45. https://doi.org/10.1016/j.cbpa.2021.01.009.

(3) Sánchez-Rico, C.; Voith von Voithenberg, L.; Warner, L.; Lamb, D. C.; Sattler, M. Effects of Fluorophore Attachment on Protein Conformation and Dynamics Studied by SpFRET and NMR Spectroscopy. *Chemistry – A European Journal* **2017**, *23* (57), 14267–14277. https://doi.org/10.1002/chem.201702423.

(4) Gomez, I. J.; Arnaiz, B.; Cacioppo, M.; Arcudi, F.; Prato, M. Nitrogen-Doped Carbon Nanodots for Bioimaging and Delivery of Paclitaxel. *J Mater Chem B* **2018**, *6* (35), 5540–5548. https://doi.org/10.1039/C8TB01796D.
